# The oxygen paradox in retinopathy of prematurity: could fetal hemoglobin be the key?

**DOI:** 10.3389/fped.2026.1844386

**Published:** 2026-06-02

**Authors:** Marcus T. A. Jackson, Ronny Amamoo, Menaka C. Thounaojam, Pamela M. Martin, Ravirajsinh N. Jadeja

**Affiliations:** Department of Biomedical Sciences, School of Graduate Studies, Meharry Medical College, Nashville, TN, United States

**Keywords:** hemoglobin, oxygenation, prematurity, retinopathy, vasculature

## Abstract

Retinopathy of prematurity (ROP) remains a leading preventable cause of childhood blindness in preterm infants. Although established risk factors such as gestational age, birth weight, and oxygen exposure are well characterized, they do not fully capture the biological variability that influences disease development. Recently emerging data suggested that the postnatal decline in fetal hemoglobin (HbF) may be an important, potentially traceable, and modifiable ROP risk factor. Lower HbF levels, particularly between 31 and 34 weeks postmenstrual age, have been associated with increased incidence and severity of ROP in infants. In addition, red blood cell transfusions from adult donors have been shown to accelerate the replacement of HbF with adult hemoglobin (HbA). This may increase retinal oxygen exposure and in turn, oxidative stress, disrupting physiologic retinal vascular development in premature infants. Mechanistically, HbF's higher oxygen affinity and redox handling properties may buffer hyperoxic injury in the premature retina. Although a multicenter randomized trial of cord blood red blood cell transfusions did not demonstrate a significant intention-to-treat benefit, analyses of protocol-adherent infants showed reduced ROP severity and treatment requirements, highlighting both therapeutic potential and practical challenges. In this perspective, we propose a precision-care approach termed “HbF stewardship,” that integrates routine HbF monitoring, transfusion optimization, selective use of cord blood products, and HbF-informed oxygen titration as a preventive strategy for ROP. We further outline potential clinical trial designs and implementation strategies to support the safe integration of hemoglobin-guided care across diverse neonatal intensive care settings.

## 1 Introduction

**Figure 1:**
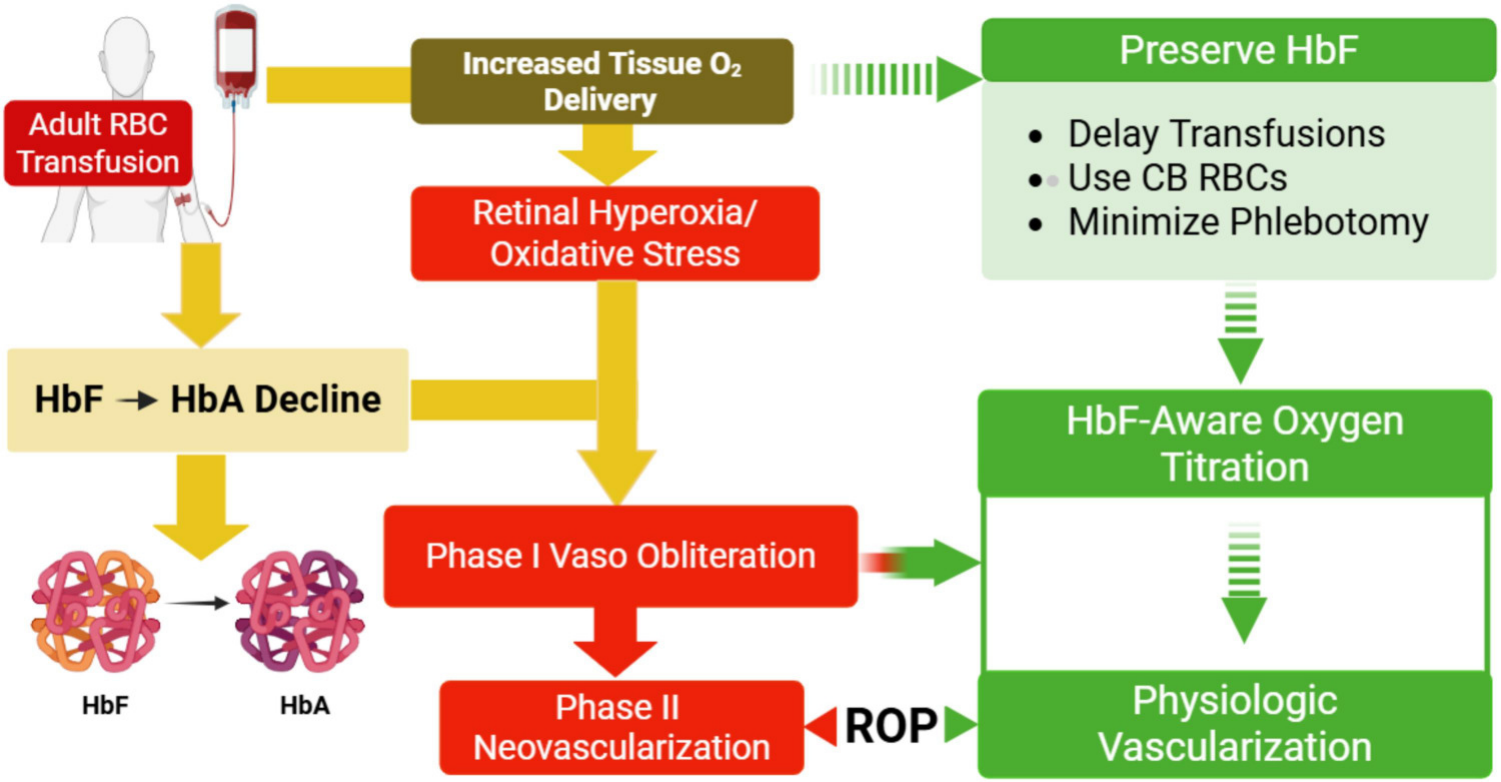
Hypothesized pathway linking transfusion timing and HbF decline to retinal hyperoxia/oxidative stress and ROP risk. Adult RBC transfusions accelerate the HbF→HbA transition; at a given SpO₂, tissue O₂ delivery increases, oxidative stress rises, and phase I vaso-obliteration and subsequent phase II neovascularization are favored. Preserving HbF (delaying transfusions, using CB-RBCs, minimizing phlebotomy) and HbF-aware oxygen titration should shift the cascade toward physiologic vascularization. Illustration was created in BioRender.

Retinopathy of prematurity (ROP) is a biphasic disorder affecting retinal vascular development characterized by early relative hyperoxia that suppresses physiologic angiogenesis (phase I), and subsequent hypoxia-driven pathologic neovascularization (phase II). Clinical strategies aimed at reducing ROP risk focus primarily on targeting oxygen exposure (1). However, landmark oxygen-targeting trials and meta-analyses have shown that lower saturation targets are associated with reduced ROP incidence but increased risks of mortality and necrotizing enterocolitis. This highlights a narrow therapeutic window and the need for more personalized approaches to oxygen delivery (2–4). Circulating hemoglobin (Hb) is an underrecognized determinant of tissue oxygen delivery in the preterm infants. Fetal Hb (HbF) predominates at birth and exhibits a left-shifted oxyhemoglobin dissociation curve relative to adult Hb (HbA), resulting in reduced oxygen unloading to peripheral tissues at a given arterial oxygen tension (5, 6). This biophysical difference may have important implications for retinal oxygen exposure during the critical early postnatal period, when antioxidant defenses are limited, and the developing retinal vasculature is particularly vulnerable to hyperoxic injury. Postnatal decline in HbF occurs rapidly in preterm infants and may be further accelerated by transfusion of adult donor red blood cells, which replaces endogenous HbF with HbA. Consequently, infants with similar peripheral oxygen saturation targets may experience substantially different retinal oxygen delivery levels depending on their hemoglobin composition, an effect not captured by current SpO₂-based oxygen management protocols (3). From this perspective, we examine HbF as a physiologic determinant of retinal oxygen exposure and propose that preserving HbF during the early postnatal vulnerability window represents a mechanism-based approach to mitigating the risk of ROP without compromising systemic oxygenation (Figure 1).

## 2 Hbf and ROP risk: consistent inverse associations

The PacIFiHER group (2021) reported that infants with HbF in the lowest tercile at 31 and 34 weeks of post-menstrual age (PMA) were between 7.6 and 12.3 times more likely to be at risk for ROP; notably, their reductions in HbF occurred before clinical ROP, which supports a causal role (7). This relationship was replicated in prospective cohorts, where lower HbF levels were associated with increased ROP and disease severity (7–12). Similarly, a multicenter Portuguese cohort (2025) reported increased RBC transfusions and reduced early HbF in affected infants with ROP, affirming HbF as a predictive biomarker in the first 4 weeks postnatal (13). Implication: HbF levels over time are useful for risk stratification and not just as indicators of illness severity (7–9, 13). Additional studies supporting this implication are highlighted in Table 1.

**Table 1:** Summary of studies examining the relationship between transfusion practices, fetal hemoglobin (HbF) levels, and retinopathy of prematurity (ROP) risk in neonates.

Study (Year)	Study Design	Gestational Age (GA)/ Body Weight (BW)	HbF Measurement & Timing	Transfusion Exposure/ HbF Replacement	Major Findings	References
Erdöl et al. (2017)	Prospective cohort (comparative); n = 49; ROP ≥ stage 1 in 26/49 (53%)	Mean GA 30.9 ± 2.7 wks (range 25–35); mean BW 1542 ± 582 g (range 520–3240)	Heel-stick samples at postnatal months 0, 1, 2, 3; HbF and HbA measured at each timepoint.	Correlation analysis included blood transfusion and HbF/HbA. Authors report transfusion significantly altered HbF and HbA levels.	Infants with ROP had lower HbF at months 1 and 2 and higher HbA at months 1 and 2. After analysis accounting for transfusion (ANCOVA), authors conclude Hb variants had no direct effect on ROP development, and transfusion was the main driver of HbF/HbA differences.	(15)
Stutchfield et al. (2017)	Prospective pilot cohort; n = 42; ROP developed in 27/62 (44%)	Mean GA: 28.0 ± 1.91 wks, Mean BW: 1042 ± 264 g	HbF measured longitudinally from postnatal blood samples during NICU stay; early postnatal HbF levels compared between infants who developed ROP and those who did not.	Infants who developed ROP had significantly greater exposure to RBC transfusions, associated with a more rapid decline in HbF due to replacement with HbA.	Infants who developed ROP had significantly lower mean postnatal HbF (∼62% vs ∼92%), independent of GA and transfusion volume. Transfusion-related HbF replacement was associated with increased ROP severity, suggesting that early HbF decline may contribute to disease development.	(14)
PacIFiHER Study Group (2021)	Prospective multicenter cohort; n = 60	Infants born at <31 weeks gestational age and/or <1500 g birth weight	HbF measured longitudinally; key assessment at 31- and 34-weeks PMA prior to clinical ROP diagnosis.	Decline in HbF over time partly associated with RBC transfusion exposure, suggesting transfusion-related HbF to HbA replacement.	Infants in the lowest HbF tertile at 31- and 34-weeks PMA had a significantly increased risk of developing ROP. Importantly, HbF decline preceded clinical disease onset, supporting HbF trajectory as a potential early physiologic biomarker of ROP risk.	(7)
PacIFiHER Study Group (2023)	Prospective multicenter cohort; n = 64;	Mean GA: 26.4 ± 1.8 weeks, Mean BW: ∼820 ± 230 g	HbF measured at birth, 31-, 34-, and 37-weeks PMA; systemic oxygenation recorded up to 42 weeks PMA.	RBC transfusion associated with decline in HbF fraction, resulting in higher proportion of HbA.	Lower HbF is associated with poorer systemic oxygenation indices and increased ROP risk. Authors note oxygen saturation targets may need to consider HbF composition.	(10)
Hellstrom et al. (2022)	Prospective observational cohort; n = 452; ROP assessed per routine screening	Mean GA: 26.4 ± 1.8 weeks, Mean BW: ∼820 ± 230 g	HbF measured longitudinally from routine blood samples during the early postnatal period, prior to clinical ROP diagnosis	Transfusion exposure evaluated in relation to HbF fraction.	Lower early postnatal HbF fraction was associated with abnormal retinal vascular development and increased risk of ROP, supporting HbF trajectory as an early physiologic marker of disease susceptibility.	(9)
Prasad et al. (2023)	Prospective observational cohort; n = 410; ROP assessed per routine screening	Mean GA: 31.3 ± 2.4 weeks Mean BW: 1456 ± 320 g	HbF measured using HPLC at enrollment and at 1-month follow-up	Blood transfusion evaluated in relation to HbF fraction	Higher HbF fraction was associated with lower prevalence and severity of ROP, independent of transfusion exposure, supporting HbF as a potential physiologic biomarker of disease risk	(8)
Prasad et al. (2023)	Prospective observational cohort; n = 410; ROP assessed per routine screening	Mean GA: 31.6 ± 2.5 weeks Mean BW: 1503 ± 330 g	HbF measured by HPLC at enrollment (baseline) and at follow-up during NICU stay	Blood transfusion exposure evaluated in relation to HbF fraction	Lower HbF levels were associated with increased risk of developing ROP and with disease progression, supporting HbF as a potential physiologic marker of ROP susceptibility.	(11)
Fevereiro-Martins et al. (2025)	Prospective multicenter cohort: ROP assessed per routine screening	Mean GA: 28.6 ± 2.4 weeks Mean BW: ∼1100 g	HbF measured from peripheral blood samples during the first 4 weeks of life	Blood transfusion exposure evaluated in relation to HbF fraction.	Lower early postnatal HbF levels were associated with increased ROP risk and severity, supporting HbF as a potential predictive biomarker during the early vulnerability window	(13)
Dani et al. (2026)	Prospective observational cohort of preterm infants; ROP assessed per routine screening	Mean GA: 29.4 ± 2.6 weeks Mean BW: ∼1250 g	HbF fraction measured from peripheral blood samples during the early postnatal period	Blood transfusion exposure evaluated in relation to HbF fraction	Lower HbF fraction was associated with increased risk of prematurity-related complications, including ROP, supporting HbF as a physiologic marker of vulnerability in preterm infants	(12)

## 3 Transfusions as a mediator of HbF decline and increased ROP risk

Red blood cell transfusions represent a clinically relevant mediator of postnatal HbF decline in preterm infants. In a prospective pilot cohort, infants who developed ROP had significantly lower mean postnatal HbF levels (∼62% vs. 92%), and this association remained significant after adjustment for gestational age, birth weight, and transfusion volume (14). Earlier work also demonstrated that infants with ROP had lower HbF and higher HbA levels in early postnatal life, while transfusion significantly influenced HbF and HbA composition, suggesting that transfusion-mediated replacement of endogenous Hb may contribute to disease risk (15). A systematic review and meta-analysis encompassing 18 studies and over 15,000 infants reported an increased risk of ROP following transfusion (pooled OR ≈1.50) (16). Importantly, both the timing and frequency of transfusions appear to influence disease risk, with early transfusions within the first 10 days of life and repeated transfusions before 28–32 weeks postmenstrual age associated with a 3–4 fold increase in the odds of severe ROP (17, 18). Transfusion of adult donor red blood cells accelerates the replacement of endogenous HbF with HbA, potentially increasing oxygen unloading to developing retinal tissue during a critical window of vascular vulnerability (14, 15).

## 4 Mechanistic plausibility: oxygen affinity and oxidative stress

Compared to HbA, HbF exhibits a higher oxygen affinity and reduced modulation by 2,3-bisphosphoglycerate, resulting in diminished oxygen unloading at equivalent arterial oxygen tensions (P50 ∼19–20 mmHg for HbF vs. ∼26–28 mmHg for HbA) (19). This left-shifted dissociation curve is advantageous *in utero*. It may also confer protection against excessive oxygen delivery to immature retinal tissue in the early postnatal period, when antioxidant defenses are limited. Consistent with this, preterm infants with lower HbF levels demonstrate increased urinary oxidative stress biomarkers, suggesting a biochemical association between HbF decline and a prooxidant state (20), and clinical studies further link reduced HbF levels to an increased risk of ROP (8–12, 14). In parallel with these studies, there is evidence linking intermittent hypoxemia/hyperoxia to oxidative damage in premature neonates and to lower hbF (21). Enhanced oxygen unloading following HbF-to-HbA replacement may increase local oxygen tension within immature retinal tissue, promoting reactive oxygen species generation in the setting of limited antioxidant defenses (5, 6, 21). Excess oxidative stress during this early postnatal period has been implicated in suppression of hypoxia-inducible factor signaling and vascular endothelial growth factor expression, thereby disrupting physiologic angiogenesis during phase I of ROP development (1). This may subsequently predispose the avascular retina to hypoxia-driven pathologic neovascularization in phase II.

## 5 Interventional signals: Can we preserve HbF to prevent severe ROP?

Can preservation of HbF during the early postnatal period help mitigate the development of severe ROP? This question has been explored in the multicenter, double-blind BORN randomized trial comparing cord blood-derived red blood cell transfusions with standard adult donor red blood cell transfusions in extremely low-gestational-age neonates (22).

Although the intention-to-treat analysis did not demonstrate a statistically significant reduction in severe ROP, per-protocol analyses excluding crossovers revealed no cases of severe or treatment-requiring ROP in the cord blood arm, compared with 34% and 26% in the adult donor group. While these findings should be interpreted with caution, they suggest that maintenance of higher HbF fractions during this vulnerable developmental window may influence disease severity (22, 23).

Contemporary oxygen-targeting trials such as NEOPROM support an SpO₂ range of approximately 90%–95% to balance mortality, necrotizing enterocolitis, and ROP risk; however, these studies did not stratify outcomes according to hemoglobin composition (24). Incorporating HbF status into oxygen titration strategies may provide an additional layer of physiologic precision, particularly in infants experiencing rapid HbF decline following transfusion, where careful avoidance of FiO₂ overshoots within established safety limits may be beneficial (2–4).

## 6 Our perspective: HbF stewardship as a pragmatic, testable framework

Converging evidence from oxygen biophysics, redox biology, and clinical epidemiology supports a plausible model in which hemoglobin composition meaningfully shapes retinal oxygen exposure in preterm infants and, in turn, may influence ROP risk alongside oxygen and transfusion practices (Figure 1). In prospective cohorts, declines in HbF occur before clinical ROP is detected, and lower HbF levels have been linked to higher oxidative stress signatures (10, 21). Broader observational data further suggest that low HbF fraction may reflect increased vulnerability to complications of prematurity, including ROP (10, 12). In addition, interventional data from CB-RBC transfusion protocols suggest that maintaining higher HbF fractions may reduce severe or treatment-requiring ROP in protocol-adherent infants, while also highlighting real-world implementation challenges (23). These observations motivate a practical strategy we term “*HbF stewardship”:* integrating routine HbF monitoring into NICU decision-making to refine transfusion and oxygen management during the early vulnerability window, to lower severe ROP risk without compromising established safety targets for survival and NEC.

Primary Anchors of HbF Stewardship: A Proposed Framework

1. ***Longitudinal HbF measurement.*** Track HbF over time (e.g., around 29–30 and 33–34 weeks PMA, and in relation to transfusion events) using standard laboratory methods such as HPLC available in most clinical settings (7, 8, 13). HbF trajectories may complement existing demographic risk factors by capturing physiologic susceptibility.
2. ***Transfusion timing and product optimization.*** Prioritize strategies that reduce avoidable early transfusions (phlebotomy reduction, restrictive thresholds where appropriate), avoid clustering of multiple early transfusions (≥3), and consider preferential use of CB-RBCs for extremely preterm infants likely to require repeated transfusions. Coordinating blood-bank workflows to reduce crossovers is essential if CB-RBC strategies are being tested in the field (17, 18, 21–23).
3. ***HbF-informed oxygen titration within consensus ranges.*** Maintain oxygen saturation targets within accepted safety ranges (approximately 90%–95% SpO₂), while recognizing that rapid HbF decline may increase tissue oxygen unloading. In this context, avoiding FiO₂ overshoots and using histogram-based analytics to maximize time-in-target may provide a practical way to reduce unnecessary hyperoxic exposure without altering guideline ranges (2–4).
  Importantly, HbF-informed oxygen stewardship is not intended to supersede established oxygen saturation targets or to imply that hemoglobin composition alone determines retinal oxygen exposure. Rather, HbF should be considered an adjunctive physiologic modifier within a multifactorial framework that encompasses prenatal hypoxia, angiogenic signaling, erythropoietic stress, transfusion burden, and oxygen instability.
4. ***Family communication and safety oversight.*** Incorporate HbF stewardship into parent-facing discussions of transfusion decisions and monitoring, using clear, standardized messaging aligned with hemovigilance principles and NICU safety practices (25).

## 7 Limitations and cautions

Residual confounding by illness severity cannot be fully excluded in observational studies examining the association between HbF and ROP risk. In addition, the intention-to-treat neutrality observed in the BORN trial underscores how logistical constraints, including product availability, compatibility requirements, and treatment crossovers, may attenuate biologically meaningful effects in real-world practice. Bedside retinal oxygenation is also not directly measurable, and although oxidative biomarker data are consistent with the proposed model, causal mediation between HbF decline, oxidative stress, and impaired vascular development remains unproven. These limitations highlight the need for implementation-focused randomized and quasi-experimental study designs to rigorously evaluate HbF-informed strategies in clinical settings (21–23).

Chronic *in utero* hypoxia, resulting from placental insufficiency, maternal smoking, preeclampsia, malnutrition, or high-altitude exposure, induces adaptive fetal responses mediated by hypoxia-inducible factor (HIF) signaling (26–28). HIF-1*α* activation may promote increased fetal hemoglobin synthesis and enhanced production of erythropoietin, insulin-like growth factor-1 (IGF-1), and vascular endothelial growth factor (VEGF). These coordinated responses may increase the HbF fraction at birth and potentially attenuate retinal oxygen unloading during the early hyperoxic phase (phase I) of retinopathy of prematurity. Additionally, hypoxia-driven erythropoietin stimulation also increases the number of circulating nucleated red blood cells (NRBCs), a biomarker repeatedly linked to increased ROP risk and severity. Elevated NRBC counts reflect heightened erythropoietic stress and have been proposed as an independent risk indicator for adverse retinal vascular outcomes (29, 30), and hence, HbF trajectories should be interpreted alongside complementary biomarkers, including NRBC burden.

Collectively, these observations suggest that prenatal hypoxia may have dual, partially opposing effects: elevated HbF may buffer oxygen-mediated retinal injury, whereas concurrent erythropoietic and angiogenic priming (via erythropoietin–HIF–VEGF signaling) may increase susceptibility to dysregulated vascular development in later phases of ROP. Infants with evidence of chronic *in utero* hypoxia may therefore constitute a biologically distinct subgroup in whom HbF trajectories should be interpreted alongside NRBC burden and other markers of prenatal hypoxic exposure, rather than in isolation.

## 8 Future directions

Future research should evaluate HbF-guided transfusion pathways using stepped-wedge or cluster-randomized trial designs that incorporate delayed first transfusion and preferential cord blood RBC use, with severe ROP and bronchopulmonary dysplasia as co-primary outcomes (22). Prespecified process endpoints, including time within target SpO₂ ranges and treatment crossover rates, will be essential for assessing implementation fidelity. Adaptive trial designs that incorporate HbF-stratified oxygen delivery within established safety bands may further determine whether HbF-informed titration can reduce severe ROP without increasing mortality or the risk of necrotizing enterocolitis, building on lessons from NEOPROM and related oxygen-targeting trials (2–4). Mechanistic cohort studies pairing longitudinal HbF trajectories with urinary oxidative stress panels and advanced oximetry or histogram analytics may help define clinically actionable HbF thresholds (3, 21). Finally, health services investigations evaluating cord blood RBC supply chains and cost-effectiveness relative to avoided laser or anti-VEGF interventions, and long-term visual impairment, are needed to inform the feasibility of broader implementation (23).

Although small-for-gestational-age infants are often presumed to reflect chronic intrauterine hypoxia, recent longitudinal data demonstrate that HbF trajectories from birth through 36 weeks post-conception may not differ significantly between small- and appropriate-for-gestational-age preterm infants, underscoring the need for direct physiologic measurements rather than assumptions based on fetal growth alone (31).

## 9 Conclusion

The integration of HbF in neonatal transfusion and oxygen management represents a biologically plausible and potentially modifiable approach to reducing the risk of severe ROP. Emerging observational and interventional evidence suggests that maintaining higher HbF fractions during the early postnatal vulnerability window may mitigate disease severity without compromising survival. Prospective trials that address logistical feasibility and implementation challenges are now required to determine whether HbF-informed care can be safely translated into routine practice in NICUs.

## Data Availability

The original contributions presented in the study are included in the article/Supplementary Material, further inquiries can be directed to the corresponding author.
